# Resection of the contrast-enhancing tumor in diffuse gliomas bordering eloquent areas using electrophysiology and 5-ALA fluorescence: evaluation of resection rates and neurological outcome—a systematic review and meta-analysis

**DOI:** 10.1007/s10143-023-02064-7

**Published:** 2023-07-27

**Authors:** David R. Peters, Floriana Halimi, Koray Ozduman, Marc Levivier, Alfredo Conti, Nicolas Reyns, Constantin Tuleasca

**Affiliations:** 1 Department of Neurosurgery, Atrium Health, Charlotte, NC USA; 2 Neurosurgery Service and Gamma Knife Center, Lausanne University Hospital (CHUV), Lausanne, Switzerland; 3 Department of Neurosurgery, School of Medicine, Acibadem Mehmet Ali Aydınlar University, Istanbul, Turkey; 4 Faculty of Biology and Medicine (FBM), University of Lausanne (UNIL), Lausanne, Switzerland; 5 IRCCS Istituto Delle Scienze Neurologiche Di Bologna, Bologna, Italy; 6 Dipartimento Di Scienze Biomediche E Neuromotorie (DIBINEM), Alma Mater Studiorum, University of Bologna, Bologna, Italy; 7 Neurosurgery and Neurooncology Service, Centre Hospitalier Regional Universitaire de Lille, Roger Salengro Hospital, Lille, France; 8 Ecole Polytechnique Fédérale de Lausanne (EPFL, LTS-5), Lausanne, Switzerland

**Keywords:** 5-ALA, Eloquent, Fluorescence, High-grade gliomas, Intraoperative neuromonitoring, Mapping

## Abstract

Independently, both 5-aminolevulinic acid (5-ALA) and intraoperative neuromonitoring (IONM) have been shown to improve outcomes with high-grade gliomas (HGG). The interplay and overlap of both techniques are scarcely reported in the literature. We performed a systematic review and meta-analysis focusing on the concomitant use of 5-ALA and intraoperative mapping for HGG located within eloquent cortex. Using PRISMA guidelines, we reviewed articles published between May 2006 and December 2022 for patients with HGG in eloquent cortex who underwent microsurgical resection using intraoperative mapping and 5-ALA fluorescence guidance. Extent of resection was the primary outcome. The secondary outcome was new neurological deficit at day 1 after surgery and persistent at day 90 after surgery. Overall rate of complete resection of the enhancing tumor (CRET) was 73.3% (range: 61.9–84.8%, *p* < .001). Complete 5-ALA resection was performed in 62.4% (range: 28.1–96.7%, *p* < .001). Surgery was stopped due to mapping findings in 20.5% (range: 15.6–25.4%, *p* < .001). Neurological decline at day 1 after surgery was 29.2% (range: 9.8–48.5%, *p* = 0.003). Persistent neurological decline at day 90 after surgery was 4.6% (range: 0.4–8.7%, *p* = 0.03). Maximal safe resection guided by IONM and 5-ALA for high-grade gliomas in eloquent areas is achievable in a high percentage of cases (73.3% CRET and 62.4% complete 5-ALA resection). Persistent neurological decline at postoperative day 90 is as low as 4.6%. A balance between 5-ALA and IONM should be maintained for a better quality of life while maximizing oncological control.

**Supplementary Information:**

The online version contains supplementary material available at 10.1007/s10143-023-02064-7.

## Introduction

Extent of resection (EOR) is associated with increased overall survival (OS) in both low-grade and high-grade gliomas (HGG) [1–7]. Similarly, increased OS is also associated with increased resection of the contrast-enhancing tumor for glioblastomas (GBM) [4, 8]. The greatest survival benefit has been observed with complete resection of the enhancing tumor (CRET) [9]. However, this survival benefit is lost if a new neurological deficit is created by microsurgical resection [10, 11]. The resection of the last 1–2% of the enhancing tumor, especially if close to eloquent areas, often carries the highest risk of neurological deficits [12]. For HGGs, minimizing the risk of new neurological deficit is especially crucial, given the limited life expectancy associated with this condition. Thus, the oncological benefit of an extended resection must be balanced with the need for sparing neurological function (maximal safe resection). This balance is particularly challenging in patients with GBM close to eloquent areas, such as the corticospinal tract (CST).

For diffuse and infiltrative gliomas, there is no clear tumor margin, complicating the process of maximal safe resection [13]. Moreover, it has been previously acknowledged by the pathologist Hans Joachim Scherer, who coined the term “neurophagie tardif,” that the infiltrated brain continues to function [14].

5-Aminolevulinic acid (5-ALA) fluorescence guidance has been developed to improve the identification of tumor margins intraoperatively, primarily with HGG, improving the extent of resection and overall survival [9, 15]. However, infiltration of tumor does not always respect functional boundaries, particularly for higher grades. Intraoperative neurophysiological monitoring (IONM) improves the understanding of functional brain borders and is crucial for the safe resection of tumors near eloquent areas. For intraoperative monitoring and mapping, direct cortical and subcortical stimulation has become the gold standard. It identifies the boundaries of eloquent brain regions and tracts that must be preserved during tumor resection to avoid neurological deficits.

For eloquent brain areas, it is not clear if adding fluorescence navigation to IONM provides a benefit, as there are scarce reports detailing the use of fluorescence in eloquent brain areas. Moreover, the interplay between increased resection with 5-ALA and preservation of function aided by IONM is not well defined.

Here, we performed a systematic review and meta-analysis of the current knowledge regarding the combined use of 5-ALA and IONM for high-grade gliomas of eloquent cortex. We review resection rates, neurological outcomes, intraoperative findings, and current recommendations.

## Methods

### Article selection and data extraction

We performed a PubMed and Embase searches for articles published between May 2006 (the seminal randomized controlled multicenter phase III trial of Stummer et al. [9]) and December 2022 using the following mesh terms: (eloquent) AND ((glioblastoma) OR (intraoperative) OR (5-ALA) OR (5-aminolevulinic)). Articles published before 2006 were excluded because they were not using the 5-ALA as an adjunct.

Inclusion criteria were as follows: patients over 18 years old, microsurgical resection of high-grade gliomas near or within eloquent brain regions, use of IONM during resection, and use of 5-ALA fluorescence guidance. Articles published in languages other than English and case reports were excluded. Table 1 illustrates the definition of adjacent to eloquent areas.

**Table 1:** Basic demographic data; data pertaining to definition of adjacent to eloquent area, intraoperative mapping technique, threshold for stopping surgery prior to complete resection

Series	Study type	Number of patients	Age (mean, range)	Sex (M:F)	Preoperative KPS (mean, range)	WHO grade (II, III, IV)	Brain region	Definition of adjacent to eloquent	Awake or asleep	Intraoperative mapping technique	Threshold for stopping surgery prior to complete resection
Feigl et al. [21] (2010)	Prospective	25 surgeries in 18 patients	55 (27–76)	12:6	90, 70–100	0, 3, 15	16 cortical, 2 subcortical	“eloquent”—not further specified	Asleep	DCS, SCS bipolar, 1 to 6 mA; TCS for MEPs, SEPs	DCS/SCS response at 1 to 6 mA,or if MEPs amplitude reduction > 50%
Della Puppa et al. [18] (2013)	Prospective	31	57 (27–79)	20:11	Med 100 (27 had 100)	0, 6, 25	19 sensorimotor, 6 right insular, 6 left language areas	< 10 mm from eloquent areas; but complete surgical resection deemed plausible on pre-op imaging assessment	25 asleep, 6 awake	DCS, SCS monopolar (asleep) bipolar (awake), 1.5 to 8 mA; EEG, ECoG for MEPs, SEPs	Functional area located prior to GTR (not further specified)
Pastor et al. [21] (2013)	Retrospective	36 surgeries in 34 patients	49.8, NR	19:15	NR, 70–100	6, 12, 18	14 cortical, 22 subcortical	“eloquent”—not further specified	Asleep	DCS, SCS monopolar; ECoG grid electrodes and/or TCS for MEPs, SEPs, VEPs	Amplitude reduction > 50% for SSEPs, VEPs, > 75% for MEPs; latencies reduced > 10% for all 3; DCS/SCS response
Schucht et al. [20] (2014)	Prospective	72	56 (22–77)	NR	80 (50–100)	0, 0, 72	NR	Adjacent to corticospinal tract (< 10 mm) on preoperative MRI; Lowest motor threshold identified intraoperatively: *N* = 8, > 20 mA *N* = 8, 11–20 mA *N* = 13, 6–10 mA *N* = 13, 4–5 mA *N* = 23, 1–3 mA	Asleep	Continuous dynamic monopolar motor mapping coupled to an acoustic motor evoked potential alarm; TCS or grid for MEPs, SEPs	Positive response at 3 mA, or sustained change in MEPs. If surgeon believed CRET was possible and MEPs stable, resection pursued to 1–2 mA
Goryaynov et al. [17] (2022)	Retrospective	34	NR	NR	NR	11, 7, 16	23 Broca, 5 Wernicke, 6 subcortical	Speech areas identified adjacent to tumor with intraoperative stimulation	Awake	Awake, 28 DCS only; 6 with DCS/SCS; TCS for MEPs, SEPs	Speech alterations at 3–4 mA or with tumor manipulation
Muscas et al. [19] (2022)	Retrospective	65	56.9, NR	30:35	NR	0, 0, 65	29 cortical, 29 subcortical, 7 deep	< 10 mm from corticospinal tract on DTI	Asleep	DCS, SCS + / − TCS or subdural electrode strips for MEPs	DCS/SCS response at 5 mA, or if MEPs amplitude reduction > 50%

*DCS* direct cortical stimulation, *SCS* subcortical stimulation, *TCS* transcranial stimulation, *MEP* motor evoked potential, *SEP* somatosensory evoked potential, *VEP* visual evoked potential, *EEG* electroencephalography, *ECoG* electrocorticography, *GTR* gross total resection, *NR* not reported

**Fig. 1:**
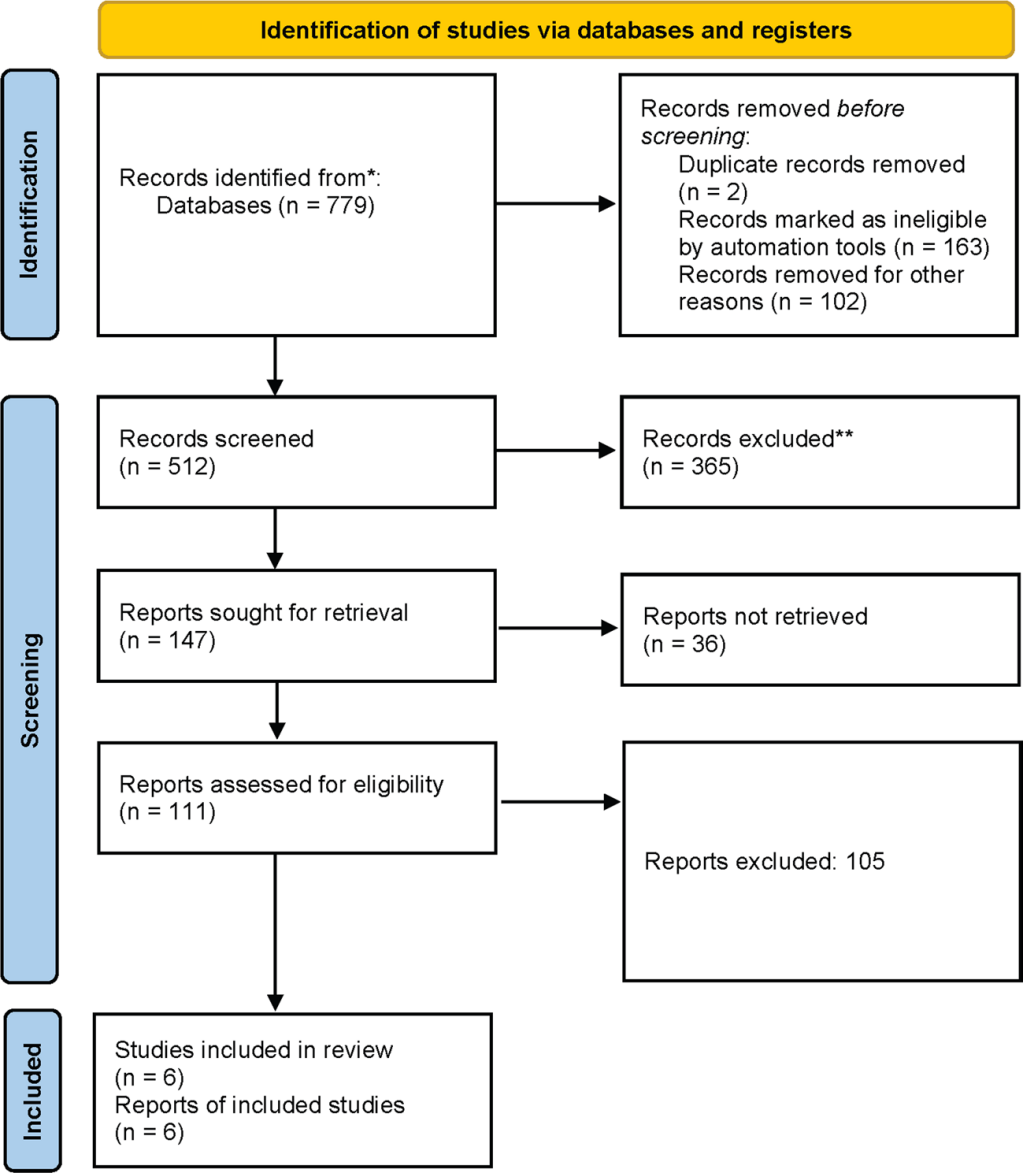
PRISMA flow chart

The present systematic review and meta-analysis was performed in accordance with the published Preferred Reporting Items for Systematic Reviews and Meta-Analyses (PRISMA) guidelines [16]. Two separate reviewers (D.P. and C.T.) applied the inclusion criteria to the search results; there were no disagreements. The article selection is exemplified in Fig. 1. Relevant biases were assessed by 2 separate reviewers (D.P. and C.T.). The present review was not registered in a systematic review database.

Were included 6 series with a total number of 254 patients undergoing 263 surgeries [17–22]. Asleep procedure was performed in 5 series [18–22], while only awake in 2 series [17, 18] (Table 1).

### 5-ALA administration procedure

5-ALA administration (20 mg/Kg 5-aminolevulinic acid orally usually 2–4 h before surgery) helps for the intraoperative detection of tumor tissue under blue-violet light [23].

### Intraoperative neuromonitoring

Electrical stimulation is particularly useful as validated intraoperative technique for identifying motor eloquent areas [24]. In the present meta-analysis, the intraoperative mapping technique varied across studies and is further detailed in Table 1. The threshold for stopping microsurgical resection prior to complete resection is further detailed in Table 1.

### Statistical analysis

In the present meta-analysis, only studies reporting individual data were selected. Because of high variations in study characteristics, a statistical analysis using a binary random-effects model (DerSimonian–Laird method) was performed using OpenMeta analyst software (Agency for Healthcare Research and Quality). Weighted summary rates were determined using meta-analytical models. Heterogeneity was tested for each meta-analysis; pooled estimates were obtained for all outcomes.

Results of series concerning the extent of resection, morbidity, and mortality were compared using a meta-regression with a random effect. *P* values < 0.05 were considered statistically significant.

## Results

### Resection rates

#### Complete resection of the enhancing tumor (CRET)

**Fig. 2:**
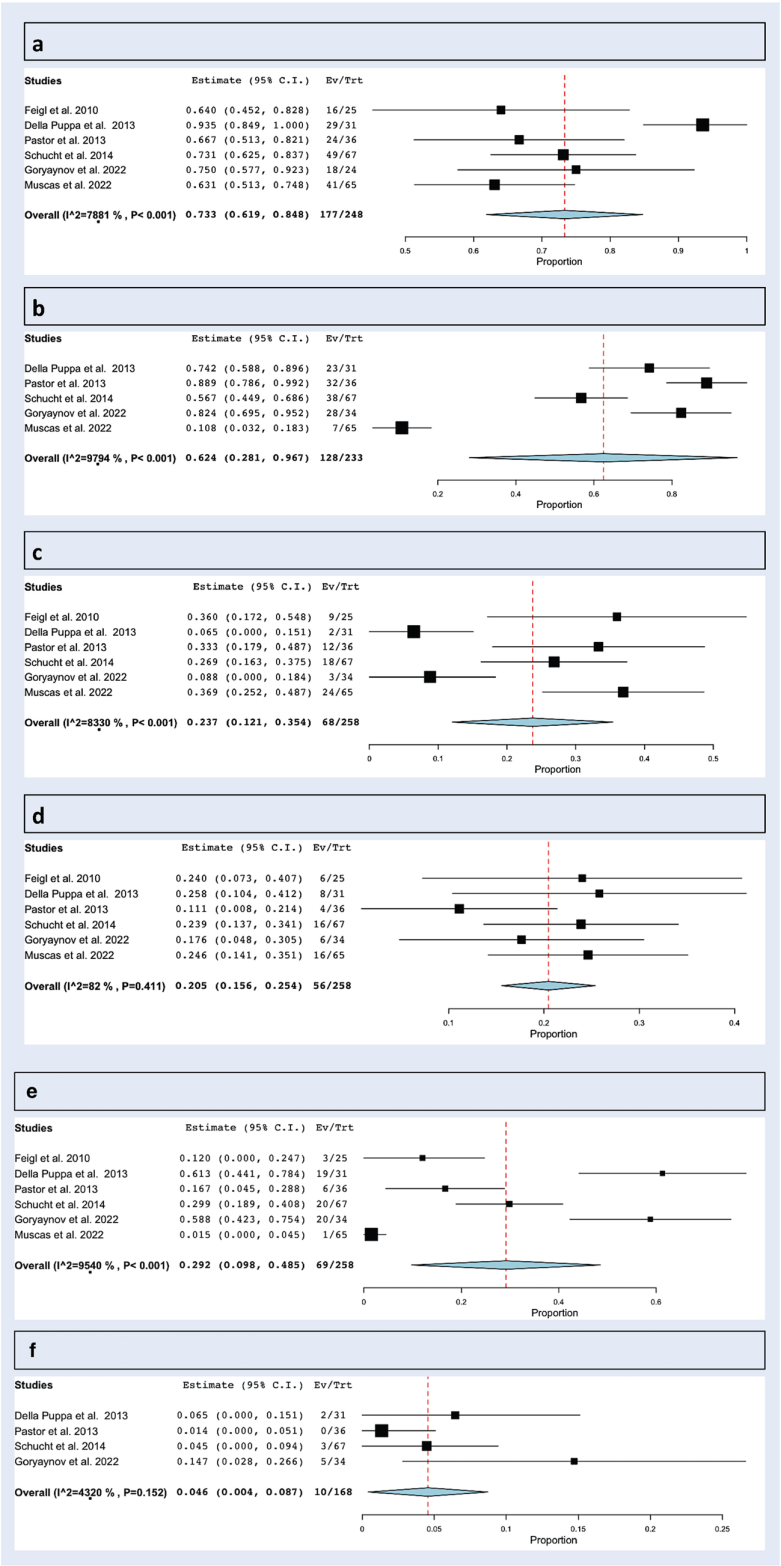
Resection rates and postoperative neurological deficits: **a** CRET; **b** complete 5-ALA resection; **c** STR; **d** resection stopped due to mapping findings; **e** neurological at day 1 and **f** at day 90

Overall rate of CRET was 73.3% (range: 61.9–84.8%, *I*^2^ = 78.81%, *p* heterogeneity < 0.001, *p* < 0.001; Fig. 2a; Table 2).

**Table 2:** Resection rates (CRET, complete 5-ALA, STR, resection stopped due to mapping findings); neurological deficit at day 1 and day 90

Series	CRET	STR	Extent of resection	Fluorescence present	Complete 5-ALA resection	Surgery stopped due to mapping findings	Preoperative neurological deficit	Neurological decline POD1	Persistent neurological deficit at day 90	Intraoperative seizure
Feigl et al. [21] (2010)	16/25	9/25	NR	25/25	NR	6/25	13/25	3/25	NR	0/25
Della Puppa et al. [18] (2013)	29/31	2/31	Over 90% all cases	31/31 bright	23/31	8/31 (all had bright residual)	6/31	19/31	2/31	1/31
Pastor et al. [21] (2013)	24/36	12/36	90.4% + / − 3.7%	NR	32/36	4/36	11/36	6/36	0/36; 10 improved, 26 stable	NR
Schucht et al. [20] (2014)	49/67 (73%)	18/67 (27%)	66/67 > 95%	67/67	38/67 (57%)	16/67 (still 9/16 had complete CRET)	22/67	20 /67 (30%)	3/67 (4%)	1/67 (1%)
Goryaynov et al. [17] (2022)	18/24 (10 did not have immediate post-op MRI)	3/24 STR, 3/24 partial resection	NR	IV: 14/16 III: 2/7 II: 4/11	28/34	6/34	NR	20/34	5/34	0/34
Muscas et al. [19] (2022)	41/65	24/65	Over 90% all cases	65/65 bright. In motor areas – 10 had none, 16 bright, 39 vague	7/65; 17 bright residual, 41 faint residual	16/65 (9 still had CRET)	47/65	1/65; 36 improved, 28 stable	NR	NR

*CRET* complete resection of enhancing tumor, *STR* subtotal resection, *NR* not reported

#### Complete 5-ALA resection

Overall rate of complete 5-ALA resection was 62.4% (range: 28.1–96.7%, *I*^2^ = 97.94%, *p* heterogeneity < 0.001, *p* < 0.001; Fig. 2b; Table 2).

#### Subtotal resection

Overall rate of subtotal resection (STR) was 23.7% (range: 12.1–35.4%, *I*^2^ = 83.3%, *p* heterogeneity < 0.001, *p* < 0.001; Fig. 2c; Table 2).

#### Surgery stopped due to mapping findings

Surgery was stopped due to mapping findings in 20.5% (range: 15.6–25.4%, *I*^2^ = 82%, *p* heterogeneity = 0.41, *p* < 0.001; Fig. 2d; Table 2).

### Postoperative neurological deficit

#### At day 1

Overall rate of neurological decline at day 1 after surgery was 29.2% (range: 9.8–48.5%, *I*^2^ = 95.4%, *p* heterogeneity < 0.001, *p* = 0.003; Fig. 2e; Table 2).

#### At day 90 (persistent)

Overall rate of persistent neurological decline at postoperative day 90 was 4.6% (range: 0.4–8.7%, *I*^2^ = 43.2%, *p* heterogeneity = 0.152, *p* = 0.03; Fig. 2f; Table 2).

## Discussion

Our systematic review and meta-analysis analyzed the combined use of 5-ALA and IONM for high-grade gliomas located within or adjacent to eloquent cortex. The overall rates for CRET, complete 5-ALA resection, subtotal resection, and surgery stopped due to mapping findings were 73.3%, 62.4%, 23.7%, and 20.5%, respectively. Immediate (day 1) postoperative deficit rates were 29.4%, while persistent at day 90 were as low as 4.6%.

### 5-ALA fluorescence

Management of high-grade gliomas within eloquent regions remains a challenge. Historically, (complete) resection of these tumors was infrequently attempted. Recently, improvements in neurosurgical adjuncts, including IONM and 5-ALA fluorescence guidance have allowed acceptable results for extent of resection, and more importantly, neurological outcome [25–27]. Previous studies have shown that fluorescence guidance with 5-ALA can define HGG tumor boundaries better than contrast-enhanced MRI, and the area of 5-ALA fluorescence is larger than the area of gadolinium enhancement for HGGs [28]. The technique utilizes filters in the operative microscope to reveal fluorescent molecules within tumor cells [27, 29, 30]. Administering 5-ALA orally before surgery enables the detection of tumor tissue during the operation under blue-violet light, which would not have been visible under white light [30]. Patients operated with 5-ALA fluorescence guidance have thus increased gross total resection rate and further recurrence-free survival [27, 31].

Approximately 90% of GBMs are fluorescence positive [32]. For non-contrast-enhancing gliomas, reports of fluorescence are much lower and range from 5 to 45% [32, 33]. For these tumors that show minimal or no fluorescence, 5-ALA is unlikely to improve the EOR. However, strong 5-ALA fluorescence in non-contrast-enhancing grades II and III gliomas most frequently represent anaplastic tumor foci [33–35]. These regions can help identify the most aggressive areas that would be the best samples for histopathological analysis, ensuring the most accurate diagnosis and allowing the best choice for targeted therapies in the era of molecular diagnosis [36].

The fluorescence intensity, typically characterized as either bright or vague, is linked to solid tumor and diffusely infiltrated regions with a positive predictive value of 100% and 97%, respectively [37]. As a general rule, bright, red fluorescent areas typically represent solid tumor and can be safely resected, although this is not universally true and attention should still be paid to IONM if the bright fluorescence is near eloquent regions [37]. Weak, vague, pink fluorescence is often a sign of tumor-infiltrated normal brain [37]. These vague areas must be handled with caution and should not be resected if the infiltrated area is eloquent.

### Neuromonitoring and 5-ALA, an appealing combination

For high-grade gliomas located within eloquent areas, fluorescence can help improve EOR but should be used in conjunction with IONM to minimize the risk of postoperative deficits. Although IONM and mapping can assist surgeons in achieving a safe tumor removal, the presence of functional tissue embedded within the tumor and/or insufficient visualization of tumor infiltration may result in a subtotal resection. Functional tissue intermixed with diffuse glioma is identified with IONM and cannot be safely resected. However, the application of 5-ALA can help overcome the challenge of inadequate visualization. That stated, systematic removal of all 5-ALA fluorescent tissue can increase the occurrence of postoperative deficits, as fluorescence may extend up to 10 mm beyond the contrast-enhancing region of the lesion on preoperative MRI, posing a risk to adjacent vital structures [38].

Completion resection of 5-ALA fluorescence without respecting the boundaries of eloquent areas carries the risk of new neurological deficits [39, 40]. The extent of resection increases survival, but new deficits cause that survival benefit to be lost and significantly reduces quality of life. It is imperative that preservation of the patient neurological function takes precedence over the extent of resection to achieve the best survival and functional outcomes.

### How close to the CST we can resect tumors

Infiltration of presumed motor eloquent areas based on preoperative MR images place patients at risk for both incomplete resection and postoperative motor deficits [24, 41]. In this respect, motor mapping to localize the CST is a useful adjunct to determine and maintain a safe distance from the CST [24, 42]. In patients with tumors adjacent or involving the internal capsule (IC) or thalamocortical fibers (TF), it is critically important to preserve these tracts to prevent permanent, worsened neurological status [21]. Thus, such high-grade gliomas in eloquent motor areas are resectable without permanent deficits once the corresponding area in question is tested negative for motor function via intraoperative mapping [24, 41–43]. It has been previously acknowledged that every 1 mA of current corresponds to 1 mm remaining distance to the CST [44].

The safe described window for monopolar high-frequency train-of-five TOF mapping is between 20 mA and 3–5 mA [44–47] or even as low as 1–3 mA [48]. Such a motor threshold (MT) excludes mechanical damage to the CST and thus prevents motor deficit, with the conditions that once stimulation becomes positive the surgeon stops the resection and that there is no vascular injury during resection.

### Sum of main conclusions as per individual series

In Table 3, we summed the main recommendations of each individual series. All authors agreed that a combination of 5-ALA, functional mapping, and neuronavigation is reliable and feasible. Resection of recurrent tumors had higher risk of neurological deficits [18]. Postoperative outcome was mainly dependent by the preoperative neurological status and second surgery [18]. Exclusive use of 5-ALA fluorescence alone may not be safe [18]. Continuous dynamic mapping and acoustic feedback is a useful technique [20]. Positive 5-ALA fluorescence in diffuse grade II gliomas may be predictive of a more aggressive disease course [17]. Motor function is more frequently found in vague fluorescence (60%) than into or adjacent to bright fluorescence [19].

**Table 3:** Main recommendations as identified per series

Series	Main message
Feigl et al. [21] (2010)	• Fluorescence and intraoperative monitoring: important tools with respect to resection radicality and functional preservation • Synergy can be achieved by combining 2 established methods
Della Puppa et al. [18] (2013)	• Recurrent tumors had higher risk of deficits • Combination of 5-ALA, functional mapping, and neuronavigation is reliable and feasible • Transient deficit never delayed adjuvant treatments in our series • Preoperative neurological status and second surgery were predictive of postoperative outcome • Patients operated in awake condition presented a permanent morbidity of 0 • STR was always related to the intentional stopping of resection driven by intraoperative monitoring • Exclusive use of 5-ALA fluorescence alone may not be safe, while the combination of IONM and 5-ALA may be synergistic
Pastor et al. [21] (2013)	• IONM can be helpful during surgery to maximize the tumor resection
Schucht et al. [20] (2014)	• High CRET rate can be safely achieved even in GBM in motor eloquent areas when using a combination of mapping and 5-ALA • Complementarity between the 5-ALA oncological benefit and functional benefit of mapping • Continuous dynamic mapping and acoustic feedback is a useful technique—monopolar stimulator attached to the end of the suction device
Goryaynov et al. [17] (2022)	• 5-ALA and awake speech mapping is useful to augment the extent of resection for infiltrative high-grade gliomas and for identifying foci of anaplasia in non-enhancing gliomas, while intraoperative speech mapping maintains safe limits of functional resection • Positive 5-ALA fluorescence in diffuse grade II gliomas may be predictive of a more aggressive disease course
Muscas et al. [19] (2022)	• Residual bright fluorescence was predictive of STR, absence was predicted of CRET • Bright fluorescence in functional areas associated with lower CRET • Motor function more frequently found in vague fluorescence (60%) than into or adjacent to bright fluorescence • Attention must be paid to removing faint fluorescent tissue for higher probability of injury function when operating close to motor pathway • Functional and fluorescence data (eloquent close to bright/vague interface) can help predict outcomes – chasing faint too far can cause deficit

*STR* subtotal resection, *IONM* intraoperative neuromonitoring, *CRET* complete resection of enhancing tumor

## Limitations

Half of the included studies were retrospective, with the risk of bias, particularly selection bias, inherent to all retrospective studies. Moreover, the definition of an eloquent area is not always clear and varied among studies. Some studies included small sample sizes; in this respect, their findings should be reproduced in larger cohorts. In some of the cohorts, there was a lack of early MRI. There is currently no study directly comparing IONM w/o fluorescence to IONM with fluorescence. Surgeon expertise also certainly factors into rates of resection and neurological deficit. Given all these limitations, the reported rates of resection and persistent neurological deficit should be interpreted cautiously.

## Conclusion

Maximal safe resection guided by intraoperative mapping and 5-ALA fluorescence of high-grade gliomas in eloquent areas is achievable in a high percentage of cases. CRET was achieved in 73.3% of cases and complete 5-ALA resection achieved in 62.4%. The rate of neurological decline at postoperative day 1 was 29.2% and persistent neurological deficit at day 90 was as low as 4.6%. 5-ALA can help improve resection, but it is extremely important to identify the functional limits of resection to ensure an adequate quality of life. High extent of resection can be safely achieved for high-grade gliomas of eloquent brain regions when using 5-ALA and IOMN together. There is a significant complementary benefit of 5-ALA and IONM. 5-ALA shows how far resection can be pursued to maximize oncological benefit, while IONM shows where the resection must stop to preserve neurological function. Such a balance between 5-ALA and IONM should be maintained to maximize oncological control without sacrificing quality of life.

### Supplementary Information

Below is the link to the electronic supplementary material.

Supplementary file1 (PDF 76 KB)

## Data Availability

Not applicable.
